# The Underlying Mechanisms of Diabetic Myopathy

**DOI:** 10.1155/2017/7485738

**Published:** 2017-11-07

**Authors:** Erick O. Hernández-Ochoa, Paola Llanos, Johanna T. Lanner

**Affiliations:** ^1^Department of Biochemistry and Molecular Biology, University of Maryland School of Medicine, Baltimore, USA; ^2^Institute for Research in Dental Sciences, Facultad de Odontología, Universidad de Chile, Santiago, Chile; ^3^Department of Physiology and Pharmacology, Karolinska Institutet, Stockholm, Sweden

## 1. Editorial

Late complications of diabetes affect both the quality and quantity of life, resulting in major health costs [1]. A common complication of both type 1 diabetes (T1D) and type 2 diabetes (T2D) is the failure to preserve muscle mass and function [2, 3], here referred to as diabetic myopathy [4–6]. Although often overlooked, this complication is believed to contribute to the progression of other diabetic complications and comorbidities (e.g., uncontrolled hyperglycemia, sedentarism, and obesity) based on the key role skeletal muscle plays in glucose homeostasis and locomotion [7–9]. Numerous studies have investigated the link between diabetic myopathy and diverse cellular processes [10]; however, despite the wealth of information on muscle weakness and muscle wasting [11], the specific triggering events of diabetic myopathy in patients with diabetes remain unknown. Further knowledge of the pathophysiological and molecular mechanisms involved in the onset and progression of diabetic myopathy is needed for the development of new pharmacological tools to ameliorate diabetic myopathy. The importance of this area of diabetes research was the motivation for us to develop this special issue.

This special issue includes three review articles and two original research papers, from leading and emerging scientists who study diabetic myopathy in different muscle tissues (cardiac, smooth, and skeletal) and with diverse expertise and interests, aiming to stimulate the continuing effort to understand the impact of diabetes on muscle function. These reviews and research papers represent the joint effort of 23 experts in the field, in which they examine the topic from several angles and levels ranging from translational aspects to whole tissue and single cell.

G. Barrientos and collaborators present a review about membrane cholesterol in skeletal muscle and its role in excitation-contraction coupling and glucose transport. They focus on the muscle plasma membrane network characterized by surface invaginations, also known as the transverse tubular (t-tubular) system [12]. This t-tubular system is rich in cholesterol [13], and it is critical for excitability and bidirectional transport of solutes, ions, nutrients, and metabolic waste. The authors discuss recent findings by other groups [14] and the work that they have done, regarding t-tubular cholesterol dynamics and its effects on excitation-contraction coupling and GLUT4 trafficking in normal and obese animal models [15, 16]. In obesity, the cholesterol content in the t-tubular network is further increased [16]. Interestingly, as suggested by the authors, restoring cholesterol levels and increasing GLUT4 trafficking in the t-tubular system could represent a new therapeutic avenue to ameliorate insulin resistance.

P. E. Morales et al. review the topic of muscle lipid metabolism and the role of lipid droplets and perilipins in rodents and humans. The skeletal muscle is not only important for carbohydrate metabolism, it is also crucial for the metabolism of lipids. Obesity is characterized by aberrant fat storage and increased levels of circulating lipids and fatty acids [17]. Lipotoxicity is characterized by the uncontrolled intracellular accumulation of lipids, oxidative stress, organelle damage, and autophagy [18]. Lipid droplets are intracellular depots, limited by phospholipid monolayer, and represent an important an immediate source of energy substrates [19]. Lipid droplets are localized mainly in the subsarcolemmal region or in between myofibrils abutted to mitochondria [20]. Here, the authors present insights into the mechanisms underlying lipid trafficking and its metabolism in skeletal muscle, especially focusing on the function of lipid droplets, the PLIN family of proteins and how these entities are modified during mechanical contraction, obesity, and insulin resistance.

The review by D. T. Au et al. focuses on the LDL receptor-related protein 1 (LRP1), a signaling receptor member of the low-density lipoprotein (LDL) receptor family involved in the clearance of chylomicrons from the circulation [21]. LRP1 is widely expressed in different tissues, with high expression in hepatocytes, adipocytes, fibroblasts, macrophages, and vascular smooth muscle cells, where it plays a critical role during angiogenesis and as an atheroprotective factor (i.e., protects against the formation of atherosclerosis) [22]. D. T. Au et al. discuss recent results using liver-specific LRP1 knockout mice [23]. Hepatic LRP1 inactivation resulted in defective insulin signaling, which included impaired phosphorylation of insulin receptor. These authors also summarize recent advances in LRP1 function in adipocytes, including studies using adipocyte LPR1 knockout mice that displayed delayed postprandial lipid clearance and improved glucose tolerance and resistance to high-fat diet-induced obesity [24]. The authors also highlight recent evidence in support of a link between LPR1 from epicardial adipocytes and glucose metabolism in individuals with T2D [25]. Due to the close proximity to muscle cells, intra- and intermuscular adipocytes may communicate with myofibers from skeletal, smooth, and cardiac tissues. Future work on LRP1 signaling could further our understanding of the cross talk between intramuscular adipocytes and the muscle.

E. O. Hernández-Ochoa and colleagues' contribution to this special issue concerns altered action potential-induced Ca^2+^ transients in cultured skeletal muscle fibers challenged with elevated extracellular glucose. As mentioned, patients with T1D and T2D exhibit increased muscle weakness and loss of muscle mass [2, 3, 11]. Yet, the mechanisms underlying diabetic myopathy remain unknown. Previous studies in skeletal muscle reported alterations in the excitation-contraction coupling (ECC)—a coordinated chain of cellular events that links the membrane action potential with intracellular Ca^2+^ release and activation of the contractile machinery [26, 27]. This study shows that muscle fibers cultured in elevated glucose for 48 hrs exhibit predominantly biphasic action potential-induced Ca^2+^ transients in response to single field stimulation. Thus, glucose-induced alterations in Ca^2+^ transients could play a role in the progression of muscle weakness and observed in diabetic myopathy.

The paper by M. L. Mizgier et al. investigated the effect of human myotube-derived media on glucose-stimulated insulin secretion in mice pancreatic islets and rat-isolated beta cells. The glucose homeostasis and insulin secretion are tightly regulated in our body [7–9]. The skeletal muscle accounts for 70–80% of the insulin-dependent glucose disposal in the postprandial state in humans [7]. The skeletal muscle is supposedly also involved in the regulation of insulin secretion from the pancreas via muscle humoral factors, also known as myokines [28, 29]. In this study, they monitored five myokines: IL6, IL8/CXCL8, MCP1/CCL2, fractalkine/CX3CL1, and RANTES/CCL5. They hypothesized that insulin influences the secretion of myokines, which in turn increases the glucose-stimulated insulin secretion (GSIS). Pancreatic islets incubated with conditioned media from human myotubes exposed to glucose exhibited higher GSIS than islets exposed to conditioned media from human myotubes in the absence of glucose stimulation. Furthermore, conditioned media from insulin-treated myotubes did not influence GSIS. They conclude that myokines present in conditioned media from untreated human myotubes regulate insulin secretion in mice pancreatic islets. These results support their hypothesis that myokines can influence insulin secretion.

We hope that more of our colleagues become interested in the study of diabetic myopathy and contribute to further our knowledge of the mechanisms of this understudied pathological process.
